# The inconsistent regulation of HOXC13 on different keratins and the regulation mechanism on HOXC13 in cashmere goat (*Capra hircus*)

**DOI:** 10.1186/s12864-018-5011-4

**Published:** 2018-08-23

**Authors:** Shanhe Wang, Zhixin Luo, Yuelang Zhang, Dan Yuan, Wei Ge, Xin Wang

**Affiliations:** College of Animal Science & Technology, Northwest A&F University, Yangling, 712100 Shaanxi China

**Keywords:** HOXC13, Keratin regulation, Hair follicle, Cashmere goat

## Abstract

**Background:**

During hair growth, cortical cells emerging from the proliferative follicle bulb rapidly undergo a differentiation program and synthesize large amounts of hair keratin proteins. In this process, HOXC13 is one critical regulatory factor, proved by the hair defects in HOXC13 mutant mice and HOXC13 mutant patients. However, inconsistent conclusions were drawn from previous researches regarding the regulation of HOXC13 on different keratins. Whether HOXC13 has extensive and unified regulatory role on these numerous keratins is unclear.

**Results:**

In this study, firstly, RNA-seq was performed to reveal the molecular mechanism of cashmere cycle including anagen and telogen. Subsequently, combining the sequencing with qRT-PCR and immunofluorescent staining results, we found that *HOXC13* showed similar expression pattern with a large proportion of keratins except for KRT1 and KRT2, which were higher in anagen compared with telogen. Then, the regulatory role of HOXC13 on different keratins was investigated using dual-luciferase reporter system and keratin promoter-GFP system by overexpressing HOXC13 in HEK 293 T cells and dermal papilla cells. Our results demonstrated that HOXC13 up-regulated the promoter activity of *KRT84* and *KRT38*, while down-regulated the promoter activity of *KRT1* and *KRT2*, which suggested HOXC13 had an ambivalent effect on the promoters of different KRTs. Furtherly, the regulation on HOXC13 itself was investigated. At transcriptional level, the binding sites of HOXC13 and LEF1 were found in the promoter of *HOXC13*. Then, through transfecting corresponding overexpression vector and dual-luciferase reporter system into dermal papilla cells, the negative-feedback regulation of HOXC13 itself and positive regulation of LEF1 on *HOXC13* promoter were revealed. In addition, melatonin could significantly increase the promoter activity of *HOXC13* under the concentration of 10 μM and 25 μM by adding exogenous melatonin into dermal papilla cells. At post-transcriptional level, we investigated whether chi-miR-200a could target *HOXC13* through dual-luciferase reporter system. At epigenetic level, we investigated the methylation level of *HOXC13* promoter at different stages including anagen, telogen and 60d of embryonic period. As a result, miR-200a and methylation were not regulatory factors of HOXC13. Interestingly, we found two SNPs (c.812A > G and c.929A > C) in the homeodomain of *HOXC13* that could deprive the regulatory function of HOXC13 on keratins without changing its protein expression.

**Conclusion:**

HOXC13 had an inconsistent effect on the promoters of different keratins. Two SNPs (c.812A > G and c.929A > C) in the homeodomain of HOXC13 deprived its function on keratin regulation. Besides, the negative-feedback regulation by HOXC13 itself and positive regulation by LEF1 and melatonin on *HOXC13* promoter were revealed. This study will enrich the function of HOXC13 on keratin regulation and contribute to understand the mechanism of hair follicle differentiation.

**Electronic supplementary material:**

The online version of this article (10.1186/s12864-018-5011-4) contains supplementary material, which is available to authorized users.

## Background

Cashmere goats are important economical animals as typical two-coated species. The guard hair grows from the primary follicles, while the cashmere is produced by the secondary follicles [1, 2]. Like hair follicle in human or murine, cashmere undergo life-long cyclical transformations, progressing through stages of rapid growth (anagen), regression (catagen), and relative “quiescence” (telogen) [3, 4] . However, significant interspecies differences exist limiting the translational potential of the murine hair follicle model. Critically, cashmere growth is a seasonal phenomenon under the control of photoperiod [5].

The most important theme for hair follicle cycling is that the follicle is a regenerating system. The basis for hair follicle regeneration rests in the unique follicular epithelial and mesenchymal components and their interactions [6–9]. Recently, some molecular signals such as fibroblast growth factor, transforming growth factor-β, WNT signaling pathway, sonic hedgehog, neurotrophins, and homeobox, and their interactions have been defined in hair follicle cycle [10–14].

During hair follicle development, a notable feature is the orderly expression of keratins (KRTs) and keratin-associated proteins (KAPs) [15, 16]. Keratins are the most abundant form of intermediate filament proteins that provide structural support in epithelial cells and keratinocytes and act as important markers of cell differentiation [17, 18]. Since selected keratins were understood to exert important regulatory functions beyond mechanical stability [18], mutations in keratin genes caused a diverse spectrum of skin, hair and mucosal disorders [19, 20]. Previous evidence had shown that the neuroendocrine regulation of human skin biology also extends to keratins. For example, thyrotropin-releasing hormone, thyrotropin, opioids, prolactin, and cannabinoid receptor 1-ligands profoundly modulated human keratin gene and protein expression in human epidermis and/or hair follicle epithelium in situ [21, 22]. Besides, a host of transcription factors could regulate keratins directly including LEF1, DLX3, FOXN1, and HOXC13 [23–26]. Specifically, The hair follicle defects were shown in both HOXC13 mutant mice and HOXC13 overexpression transgenic mice [27, 28], which suggested that HOXC13 had a critical regulatory role on keratin expression during hair follicle development. Meanwhile, HOXC13 was also considered as a crucial regulator of murine hair cycle [29]. Further studies showed HOXC13 was involved in the regulation of human hair keratin gene expression [23, 30], in which Tkatchenko and his colleagues revealed that overexpression of HOXC13 in differentiating keratinocytes resulted in the down regulation of a novel hair keratin gene cluster [30], while Jave-Suarez showed that HOXC13 strongly activated the promoters of the keratin genes. Obviously, the previous conclusions of the effect of HOXC13 on different keratins were inconsistent. Whether HOXC13 has extensive and unified regulatory role on these numerous keratins was still unclear.

*HOXC13* gene (NC_030812) is composed of two exons spanning 7.829 kbs of genomic DNA, encoding a conserved protein of 330 amino acids. It contains a cluster of 61 amino acids (amino acids 258–318) forming the DNA binding homeodomain [31]. This homeodomain is involved in the regulation of transcriptional activity of several hair keratin genes and *FOXN1* in hair follicles and nails [32]. To date, seven mutations had been reported in this gene causing pure hair and nail ectodermal dysplasia (PHNED), which led to complete hair loss and nail dysplasia (Table 1) [33–35]. Interestingly, two single nucleotide polymorphisms (SNPs) (c.812A > G and c.929A > C) in the homeodomain that belong to missense mutation caused ectodermal dysplasia [36, 37]. However, the mechanism was still unknown.

**Table 1:** Summary of Mutations in HOXC13 in Families with Pure Hair and Nail Ectodermal Dysplasia

Author	Nucleotide change	Amino acid change	Ethnicity	Zygosity	Consanguinity
Lin et al., 2012 [33]	c.390C > A	p.Tyr130*	Chinese	Homozygous	Consanguineous
Lin et al., 2012 [33]	Microdeletion (chr12: 54,308, 194–54, 335, 815)	Deletion of exon 1	Afghan	Homozygous	Consanguineous
Farooq et al., 2013 [35]	c.355delC	p.Leu119Trpfs*20	Syrian	Homozygous	Consanguineous
Ali et al., 2013[34]	c.200-203dupGCCA	p.His68Glnfs*84	Pakistani	Homozygous	Consanguineous
Ali et al., 2013[34]	c.404C > A	p.Ser135*	Pakistani	Homozygous	Consanguineous
Li et al., 2017[37]	c.812A > G	p.Gln271Arg	Hispanic	Homozygous	Consanguineous
Khan al, 2017[36]	c.929A > C	p.Asn310Thr	Pakistani	Homozygous	Consanguineous

In conclusion, HOXC13 play a critical role in hair follicle development and keratin regulation. However, the regulatory function of HOXC13 on different keratins is still incomplete. Besides, the regulation mechanism on HOXC13 is also poorly understood. To explore above questions, in this study, RNA-seq was performed to reveal the molecular mechanism of cashmere cycle. We found that *HOXC13* had similar expression trend with a large proportion of *KRTs* and *KAPs* in anagen and telogen. Then, the inconsistent effect of HOXC13 on different keratins was detected using dual-luciferase reporter system and keratin promoter-GFP system. Finally, the regulation mechanism on HOXC13 was explored at transcriptional, post-transcriptional and epigenetic levels. This study will enrich the function of HOXC13 on keratins regulation and contribute to understand the mechanism of cashmere development and differentiation.

## Methods

### Animals

Shanbei cashmere goats with the fine fiber production trait were used in this study. All the goats were obtained from Shanbei cashmere goats engineering technology research center of Shaanxi province, China. The experimental animals were fed according to the local Cashmere goat standard of Shaanxi (DB61/T583–2013, http://www.sxny.gov.cn/). Six female adults (one year old, coefficient of relationship < 0.125) were selected to obtain skin samples at anagen and telogen. After infiltration anaesthesia through hypodermic injection of 2% lidocaine hydrochloride (100 mg), skin samples approximately 2 cm^2^ and 3 mm deep were harvested from the body side of adult goats at distinct hair cycle stages (anagen and telogen), frozen in sample protector for RNA (Takara, China) and stored at − 80 °C for future analysis. The same animals were collected at an adjacent position at both anagen and telogen. The animals will recover in two weeks with proper care.

Six pregnant Shanbei White cashmere goats (two years old, weighing 30–40 kg) at the same stage of pregnancy were selected to obtain fetal skin samples at 60^th^d of embryonic period (E60) and E120. Each time point had three replicates. E60 represented the initiation stage of hair follicle morphogenesis, and E120 represented the development stage. After intravenous injection of Rompun (0.3 mg/kg) for anesthesia, six fetuses were delivered from six different females by caesarean operation. Proper care were made up for the ewes after the operation, while the fetuses died soon after cutting umbilical cord. Skin samples from fetuses were collected from the right mid-side of the fetus, rinsed in ice-cold DEPC-treated water and cut into small pieces. Every skin sample was divided into two parts; one was fixed with 4% paraformaldehyde and one was frozen in sample protector for RNA (Takara, China) and stored at − 80 °C for qRT-PCR analyses. The carcasses were frozen to designated location waiting bio-safety disposal.

All the experimental procedures with goats used in the present study had been given prior approval by the Experimental Animal Manage Committee of Northwest A&F University (2011–31,101,684). All the operations and experimental procedures were complied with the national standard of Laboratory animal-Guideline for ethical review of animal welfare (GB/T 35892–2018) and Guide for the Care and Use of Laboratory Animals: Eighth Edition [38].

### RNA isolation, library preparation, sequencing and bioinformatics analysis

Total RNA was extracted from the collected skin tissues using Trizol reagent (Invitrogen, USA) following the manufacturer’s instructions, after grinding them in liquid nitrogen. The RNA concentration and quality was determined using the Agilent 2100 Bioanalyzer (Agilent Technologies, USA). The extracted total RNA was stored at − 80 °C for later use.

For transcriptome sequencing and bioinformatics, the specific methods were presented in our previous paper [39]. Briefly, a total amount of 3 μg RNA per sample was used as input material for the RNA library preparations. Firstly, ribosomal RNA was removed using the Epicentre Ribo-zero™ rRNA Removal Kit (Epicentre, USA), and the rRNA was cleaned up by ethanol precipitation. Subsequently, in total six libraries from anagen (*n* = 3) and telogen (*n* = 3) were generated from the rRNA-depleted RNA using the NEBNext® Ultra™ Directional RNA Library Prep Kit for Illumina® (NEB, USA) following the manufacturer’s recommendations. Strand-specific sequencing was performed on the Illumina Hiseq 4000, PE 150 system for these libraries (paired-end 100-bp reads). Raw data were first processed using in-house Perl scripts. The high quality reads were mapped independently to the goat genome v2.0 (ftp://ftp.ncbi.nlm.nih.gov/genomes/all/GCA/000/317/765/GCA_000317765.2_CHIR_2.0) using Bowtie v2.0.6 [40] and the spliced read aligner TOPHAT v2.0.9 (main parameter: library-type <fr- firststrand>) [41]. The mapped reads of each sample were assembled using Cufflinks (v2.1.1) in a reference-based approach [42]. Cufflinks was run with ‘min-frags-per-transfrag = 0’ and ‘--library-type’, other parameters were set as default. Cuffdiff (v2.1.1) [42] was used to calculate fragments per kb per million reads (FPKM) of genes in each sample. It was also used to provide statistical routines for determining differential expression in gene expression data using a model based on the negative binomial distribution. Transcripts or genes with a *P*-adjust ≤0.05 [43] and fold change ≥2 were described as differentially expressed between anagen and telogen.

Gene Ontology enrichment analysis of differentially expressed genes was implemented using Gene Ontology Consortium (http://www.geneontology.org/) [44]. Gene ontology terms with corrected *p* value less than 0.05 were considered significantly enriched by differentially expressed genes. Pathway analysis was used to identify significant pathways for the differentially expressed genes according to the Kyoto Encyclopedia of Genes and Genomes (KEGG) (http://www.genome.jp/kegg/) [45]. We used KOBAS software (main parameter: blastx 1e-10; padjust: BH) to test the statistical enrichment of differentially expressed genes in KEGG pathways [46]. The microRNAs that may target HOXC13 were predicted by TargetScan (http://www.targetscan.org/vert_71/). The tertiary structure of HOXC13 was predicted by Phre2 (http://www.sbg.bio.ic.ac.uk/phyre2/).

### Quantitative real-time PCR (qRT-PCR)

The total RNAs for RNA-seq were also used for quantitative PCR analysis. For mRNAs, the first-strand cDNA was obtained using a PrimeScript™ RT reagent Kit with gDNA Eraser (TAKARA, China), and then were subjected to quantification of the mRNAs with β-actin as an endogenous control on the Bio-Rad CFX96 Touch™ Real Time PCR Detection System (Bio-Rad, USA). The qRT-PCR reaction consisted of 10 μL 2 × SYBR® *Premix Ex Taq*™ II (TAKARA, China), 0.8 μL specific forward/reverse primer (10 μM), 1 μL cDNA, and ddH_2_O to a final volume of 20 μL. The quantitative PCR was performed using the following conditions: 95 °C for 60 s, 40 cycles of 95 °C for 10 s, and the optimized annealing temperature for 30 s. Each stage (anagen and telogen) included at least 3 samples, and all reactions were performed in triplicate for each sample. The primers and annealing temperatures for genes were listed in Additional file 1.

For chi-miR-200a, the first-strand cDNA was obtained using stem-loop RT primer provided by Hairpin-it™ microRNA and U6 snRNA normalization RT-PCR quantitation Kit (GenePharma, China), then its expression was detected by qRT-PCR using Taqman probe provided by the above Kit after transfecting the mimics and inhibitor into dermal papilla cells. The qRT-PCR reaction consisted of 10 μL 2 × Real time PCR Mix (FAM), 0.4 μL miR-200a/U6-specific primer set (10 μM), 0.3 μL miR-200a/U6-specific probe (10 μM), 2 μL miRNA RT product, 0.2 μL Taq DNA polymerase and ddH_2_O to a final volume of 20 μL. The quantitative PCR was performed using the following conditions: 95 °C for 180 s, 40 cycles of 95 °C for 12 s, and 62 °C for 40 s on the Bio-Rad CFX96 Touch™ Real Time PCR Detection System. U6 was used as an endogenous control. The primers for miR-200a and U6 were unknown as trade secret.

Gene expression was quantified relative to the endogenous gene expression using comparative cycle threshold (ΔCT) method [47] through Bio-Rad CFX Manager 3.1 and Microsoft excel 2013. Differences in gene expression between the groups were detected by independent sample *t*-test.

### Immunohistochemistry (IHC)

IHC were performed as previously described [48] with little modifications. Briefly, skin samples from different stages including anagen, telogen, E60 and E120 were fixed with 4% paraformaldehyde at 4 °C overnight, followed by dehydration procedure in gradient ethanol series. The samples were then rinsed with xylene and further embedded in paraffin. Embedded samples were serially cut into 5 μm sections with a microtome (Leica RM2255, Nussloch, Germany). Before staining, slides were deparaffinized in xylene and antigen retrieval was performed in 0.01 M sodium citrate buffer at 96 °C for 10 min. PBS supplemented with 10% goat serum and 3% bovine serum albumin (Sigma, USA) was used for blocking at room temperature for 40 min. Primary antibody against HOXC13 (SAB, USA) was then incubated with the samples at 4 °C overnight. Goat anti-rabbit Ig-CY3-conjugated secondary antibody (Beyotime biotechnology, China) were used to specifically bind to primary antibody at 37 °C for 30 min. Hoechst33342 (Beyotime biotechnology, China) was used for nuclei staining and the slides were finally mounted with Vecatshield mounting media (Vector, USA). All pictures were taken under Olympus BX51 fluorescence microscope imaging system (Olympus, Japan).

### Plasmid construction

In order to seek the optimal promoters of specific *KRTs* and *HOXC13*, promoters of *KRTs* and *HOXC13* with different length were amplified by PCR using goat genomic DNA as template (for PCR primers, see Additional file 1). The PCR products were cloned into the PGL3-basic vector using *Xho* Ι/*Nhe* Ι and *Kpn* Ι enzymes. The vectors were named as *KRT1*–362 and so on. In order to verify the binding site of HOXC13 in the promoter of HOXC13 itself, the predicted binding site (TT(A/T)ATNPuPu) in the optimal promoter of *HOXC13* (HOXC13P365) was mutated by introducing a *EcoR* Ι recognition site (HOXC13P365 mut). The primers were listed in Additional file 1.

In order to investigate the function of HOXC13 in vivo, the overexpression vector of HOXC13 were constructed. Briefly, the full length ORF of *HOXC13* was amplified by PCR using goat skin cDNA as template. The PCR products were cloned to pcDNA(+)3.1 vector (Invitrogen, USA) using *EcoR* Ι and *Kpn* Ι (wtHOXC13). As control vector, the ORF without homeodomain was amplified and constructed into pcDNA(+)3.1 using goat genomic DNA as a template (ΔHOXC13). For site specific mutational overexpression vector of HOXC13, the site specific mutation of full length ORF was amplified by overlap-PCR using the WT vector as template (HOXC13 812mut, HOXC13 929mut). *KRT* promoter-GFP vectors were constructed to furtherly reveal the effect of HOXC13 on the promoters of *KRTs* visually. The CMV promoter before GFP was taken place by the promoter of *KRTs* using *Not* Ι and *BamH* Ι. For KRT2P–GFP, the promoter was amplified using the primers listed in the Additional file 1. For KRT84P-GFP, the promoter of *KRT84* was cut from the vector of *KRT84* promoter-PGL3 basic using *Not* Ι and *BamH* Ι. The basic vector (JMB84-T2A-EGFP) was donated by prof. Zhiying Zhang (Additional file 3: Figure S1).

LEF1 overexpression vectors were constructed using pcDNA(+)3.1 vector through *EcoR* Ι and *Kpn* Ι enzymes. The different spliceosomes of *LEF1* (*LEF1* X1, *LEF1* X3) were amplified and purified using the same primers, while the vector with truncated *LEF1* was constructed using the other reverse primer as a negative control (ΔLEF1). The primers were listed in Additional file 1.

In order to explore the regulation mechanism of miR-200a on HOXC13 expression, the *HOXC13* 3′-UTR segment, containing miR-200a binding sites, was amplified. Then the amplified segment was implanted into the psiCHECK2 vector to construct psiCHECK2-HOXC13–3′-UTR plasmid.

### Cell culture and transfection

HEK 293 T cells were cultured in Dulbecco’s modified Eagle’s medium (DMEM) Gibco, USA) with 10% fetal bovine serum (FBS) (Gibco, USA), 1% Antibiotic-Antimycotic (Gibco, USA) at 37 °C with 5% CO_2_.

For dermal papilla cells, skin sample was collected from the dorsal skin and immediately rinsed in ice-cold saline supplemented with 100 U/mL penicillin and 100 mg/mL streptomycin (Gibco, USA), then the samples were clipped with scissors, and ophthalmic forceps (Sigma, USA) were used to isolate goat secondary hair follicles. For dermal papilla cell in vitro isolation, single secondary hair follicles were trypsinized with 0.25% trypsin-EDTA solutions at 37 °C for 15 min, then the hair bulb was mechanically cut down with a 1 mL syringe and the obtained tissues were cultured with dermal papilla cell media in adherent culture dishes. The dermal papilla media consisted of DMEM/F12 media supplemented with 10% FBS and 2% B27 supplement (Gibco, USA), 20 ng/mL EGF (Sigma, USA), 40 ng/mL recombinant human FGF-basic (bFGF, PeproTech, USA) and 1% Antibiotic-Antimycotic. Trypsinization purification approach was used to remove fibroblasts.

For seeking the optimal promoter of specific *KRTs* and *HOXC13*, the corresponding *KRT* promoter-pGL3 basic vector/ *HOXC13* promoter PGL3 basic vector together with the reference vector pRL-TK were co-transfected into 24-well dermal papilla cells using Lipofectamine 2000 according the manufacture protocol (Invitrogen, USA), while the empty vector of PGL3 basic was used as a negative control. All the groups were performed in triplicate. For investigating the regulatory role of HOXC13 on different *KRTs*, the corresponding *KRT* promoter-pGL3 basic vector, pRL-TK vector together with HOXC13 overexpression vector (wtHOXC13/ΔHOXC13) were co-transfected into 12-well dermal papilla cells and HEK 293 T cells using Lipofectamine 2000, while the vector of ΔHOXC13 was used as a negative control. Subsequently, the vector of *HOXC13* mut was used as another treatment group. To verify the results visually, *KRT* promoter-GFP system was used. The corresponding *KRT* promoter-GFP vector together with HOXC13 overexpression vector (wtHOXC13/ΔHOXC13) were co-transfected into 12-well HEK 293 T cells while the vector of ΔHOXC13 was used as a negative control. Similarly, *KRT* promoter-GFP vector together with *HOXC13* mut overexpression vector was used to investigate the effect of the SNPs on keratin regulation in HEK 293 T and dermal papilla cells. To investigate the effect of the two SNPs on HOXC13 expression, HOXC13wt / HOXC13 812mut / HOXC13 929mut vector was transfected into HEK 293 T cells, respectively. To investigate the effect of HOXC13 and LEF1 on the promoter activity of *HOXC13*, HOXC13/LEF1 overexpression vector together with HOXC13 p365-PGL3 basic vector were transfected into dermal papilla cells, while ΔHOXC13 / ΔLEF1/ HOXC13 mut was used as a negative control. HOXC13P365 mut was a negative control to verify the binding site of HOXC13 in the promoter of HOXC13 itself. To verify the interaction of chi-miR-200a and *HOXC13* 3′ UTR, chi-miR-200a mimics/inhibitor/ the corresponding negative control and HOXC13 3′ UTR -psiCHECK2 were transfected into HEK 293 T cells, respectively. All the groups were performed in triplicate.

### Dual-luciferase assay

About 48 h post-transfection, cells were harvested and lysed in passive lysis buffer (Promega, USA). Lysates were assayed for reporter gene activity with dual-luciferase assay system (Promega, USA) according to the manufacturer’s instruction. For PGL3 basic system, the firefly luciferase signal was normalized to the renilla luciferase signal. For psi-CHECK2 system, the renilla luciferase signal was normalized to the firefly luciferase signal. The normalized firefly/renilla luciferase activity was compared using a student’s t-test (*p* < 0.05).

### Western blotting

The total protein was extracted from transfected HEK 293 T cells, blank HEK 293 T cells and skin sample from mouse using RIPA cell lysis solution (Applygen Technologies Inc., China), while blank cells and the mouse skin sample were used as a positive control. The samples were separated on a 10% SDS-PAGE gel (30 μg protein per sample) and transferred onto nitrocellulose membranes (Bio-Rad, USA). After blocking with 5% skimmed milk powder solution for 1 h at 37 °C, the membranes were incubated overnight at 4 °C with a murine polyclonal rabbit-antibody against HOXC13 and GAPDH (Signalway Antibody, USA), then incubated with a HRP conjugated secondary goat-anti-rabbit IgG (Zhongshan-Bio, China). The protein bands were visualized with Super ECL Plus (ApplyGEN, China). And GAPDH was taken as a reference control.

### Melatonin

Melatonin was obtained from Sigma Chemical Co. (Sigma, USA). For the purposes of investigating its effect on HOXC13 promoter, dermal papilla cells were transfected with HOXC13-promoter-PGL3 basic vector and /without melatonin in pharmacological (1–25 μM) concentrations.

### MiR-200a mimics and inhibitor

Chi-miR-200a mimics, inhibitor and its negative control (NC) oligonucleotides were synthesized by Shanghai GenePharma Co., Ltd. (Shanghai, China). The oligonucleotide sequences were listed in Additional file 1.

### Bisulphite sequencing polymerase chain reaction (BSP)

Genomic DNA of skin samples including different stages (anagen, telogen and E60) was extracted following the standard procedures using TIANamp Genomic DNA Kit (Tiangen, China). Every stage included 3 biological repetition. DNA treatment with sodium bisulphite was performed using the EZ DNA Methylation Kit (Zymo Research, USA) according to the manufacturer’s protocol, except that the conversion temperature was changed to 55 °C. The modified DNA samples were diluted in 10 μL of distilled water and should be immediately used in BSP or stored at − 80 °C until PCR amplification. The BSP primers were designed by the online MethPrimer software (http://www.urogene.org/methprimer/). The sequences of PCR primers used for amplifying the targeted products were shown in Additional file 1. We used hot start DNA polymerase (Zymo Taq ™ Premix, Zymo Research, USA) for BSP production. PCR was performed in 50 μL of reaction volume, containing 200 ng genomic DNA, 0.3 μM of each primer, Zymo Taq ™ Premix 25 μL. The PCR was performed with a DNA Engine Thermal Cycler (Bio- Rad, USA) using the following program: 10 min at 95 °C, followed by 45 cycles of denaturation for 30 s at 94 °C, annealing for 40 s at 52 °C and extension for 30 s at 72 °C, with a final extension at 72 °C for 7 min. The PCR products were gel purified using Gel Purification Kit (Sangon, China), and then subcloned into the pGEM T-easy vector (Promega, USA). Different positive clones for each subject were randomly selected for sequencing (Sangon, China). We sequenced 4 clones from each independent set of amplification and cloning, hence, there were 12 clones for the HOXC13 DMR in each stage. The final sequence results were processed by online QUMA software15 (http://quma.cdb.riken.jp/top/index.html).

## Results

### RNA-seq to reveal the molecular mechanism of cashmere cycle

**Fig. 1:**
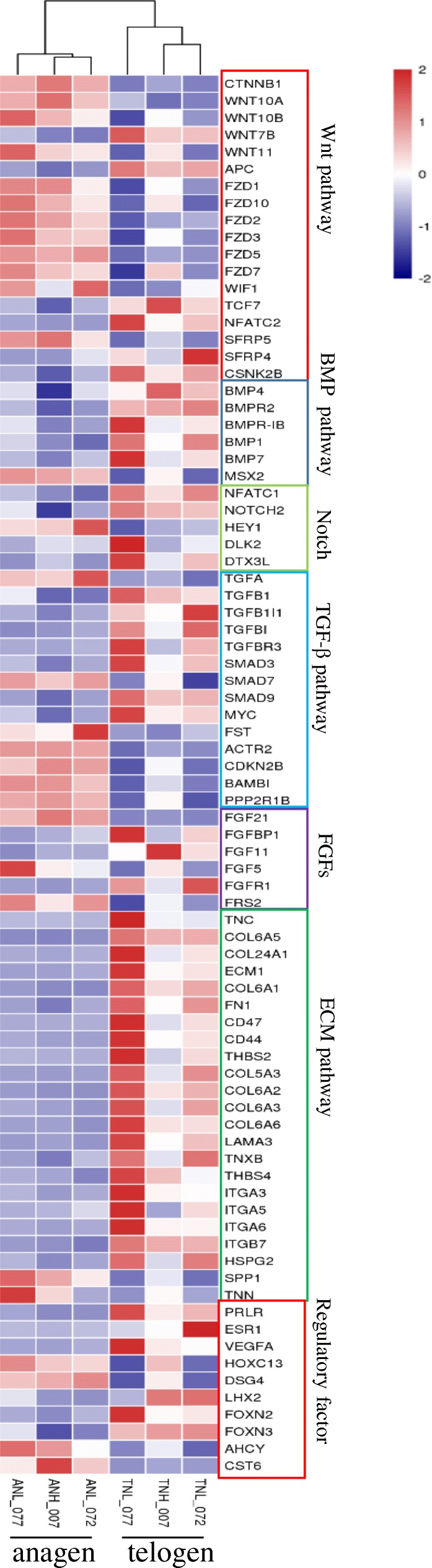
The differentially expressed genes and their enriched pathways which are critical in hair follicle cycling were shown in Heat Map. Wnt, BMP, TGF-β, ECM pathways, FGFs and other important regulatory factors related in hair follicle biology were shown according previous studies on hair follicle biology. Genes belong to one pathway were located in one frame

To gain insights into molecular mechanism of cashmere cycle, we previously performed transcriptome sequencing using skin samples at anagen and telogen. A total of 644,884,566 raw reads were generated using the Illumina HiSeq 4000 Platform in which 623,168,524 reads were clean. The generation rate of clean reads was higher than 85% for all samples. A total of 2492 differentially expressed mRNAs (Fold Change ≥2 and P-adjust value ≤0.05) were identified between anagen and telogen [38]. Among them, the genes and pathways which are critical in hair follicle cycling were selected according previous study on hair follicle biology (Fig. 1). To better understand the gene networks involved in cashmere cycling, gene ontology and KEGG analyses were performed. The gene ontology categories of differentially expressed genes included cell-cell adhesion, development growth, positive regulation of cell migration and regulation of differentiation (Additional file 2), consistent with an increase of differentiation and proliferation during hair matrix and hair shaft formation. KEGG analysis predicted that the differentially expressed genes were enriched in 274 pathways. Among the identified KEGG pathways, some belonged to conventional pathways associated with hair follicle cycling and hair shaft formation, such as the WNT signaling pathway, ECM-receptor interaction, TGF-β signaling pathway and VEGF signaling pathway.

### Spatio-temporal expression pattern of HOXC13 during cashmere morphogenesis and postnatal cycle

**Fig. 2:**
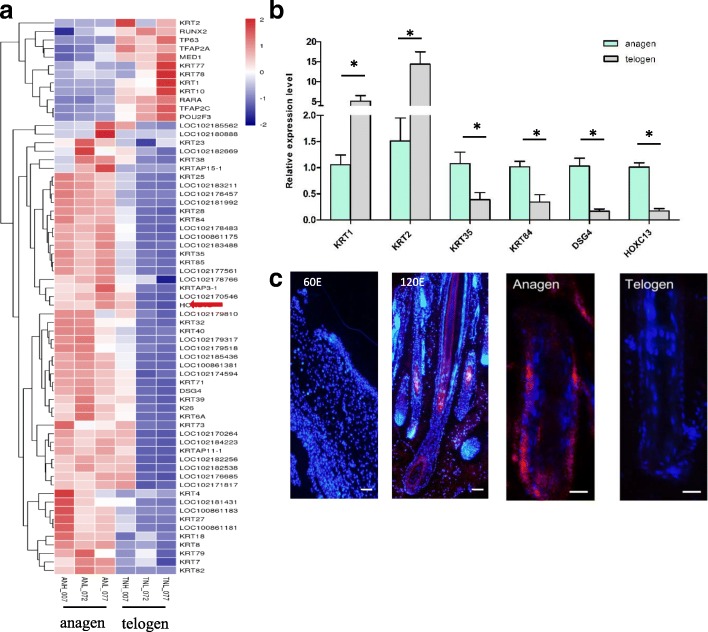
*HOXC13* had a similar expression pattern with the majority of *KRT*s in anagen and telogen. **a** Differentially expressed *KRT*s and related regulatory genes were shown in Heat Map. *HOXC13* had similar expression trend with the majority of KRTs, which were markedly up-regulated in anagen compared with telogen. **b** qRT-PCR was performed on selected *KRT*s, *HOXC13* and *DSG4* to verify the sequencing data. *HOXC13*, *DSG4*, *KRT38* and *KRT84* were up-regulated, while *KRT1* and *KRT2* were down-regulated in anagen compared with telogen. The expression of specific gene was quantified relative to the expression level of *β-actin* using the comparative cycle threshold (ΔCT) method. The data was expressed as the mean ± 1 SE (*n* = 3). * *P* < 0.05. **c** Immunostaining of HOXC13 in cashmere goat hair follicles at embryonic period (65d and 120d), anagen and telogen. Red fluorescence indicated the nuclear expression pattern of HOXC13. Nucleus was stained with Hoechst in blue

It was noteworthy that our analysis identified a set of genes belonging to *KRTs* and *KAPs*, which were markedly up-regulated in anagen compared with telogen. Meanwhile, we found *HOXC13* gene, one critical regulatory factor involved in hair follicle development, which had similar expression trend with these *KRTs* and *KAPs* (Fig. 2a). The result suggested that there might be a link between HOXC13 and KRTs. Interestingly, *KRT1* and *KRT2* were exceptions among these *KRTs,* which had opposite expression pattern with *HOXC13*. The result indicated that HOXC13 had different effect on *KRT1* and *KRT2* compared with other *KRTs*. Other genes involved in keratin regulation such as *RUNX2*, *tp63*, *MED1* were also shown in Fig. 2a.

In order to verify *HOXC13* and *KRTs*′ expression profiles in anagen and telogen, qRT-PCR was performed on selected *KRTs*, *HOXC13* and *DSG4* (another regulatory factor involved in keratin regulation). As a result, the qRT-PCR data was in concordance with the RNA-seq data. Briefly, *HOXC13*, *DSG4*, *KRT38* and *KRT84* were upregulated in anagen (*P* < 0.05), while *KRT1* and *KRT2* were downregulated in anagen compared with telogen (*P* < 0.05) (Fig. 2b). Furtherly, IHC was performed to show HOXC13 expression in different hair follicle development stages including embryonic period (E60 and E120) and postnatal period (anagen and telogen) at protein level. HOXC13 was intensively expressed in the outer root sheath, inner root sheath, matrix and medulla cells of hair follicles in a nuclear expression pattern at the stage of E120 and anagen, while it was not detected at the period of E60 and telogen (Fig. 2c). Consistent with that hair shaft is growing rapidly in E120 and anagen during which keratins are expressing actively and intensely, while is not growing in E60 and telogen. The result further indicated that HOXC13 was involved in the regulation of keratins in Cashmere goat.

### HOXC13 have an inconsistent effect on the promoters of different KRTs

**Fig. 3:**
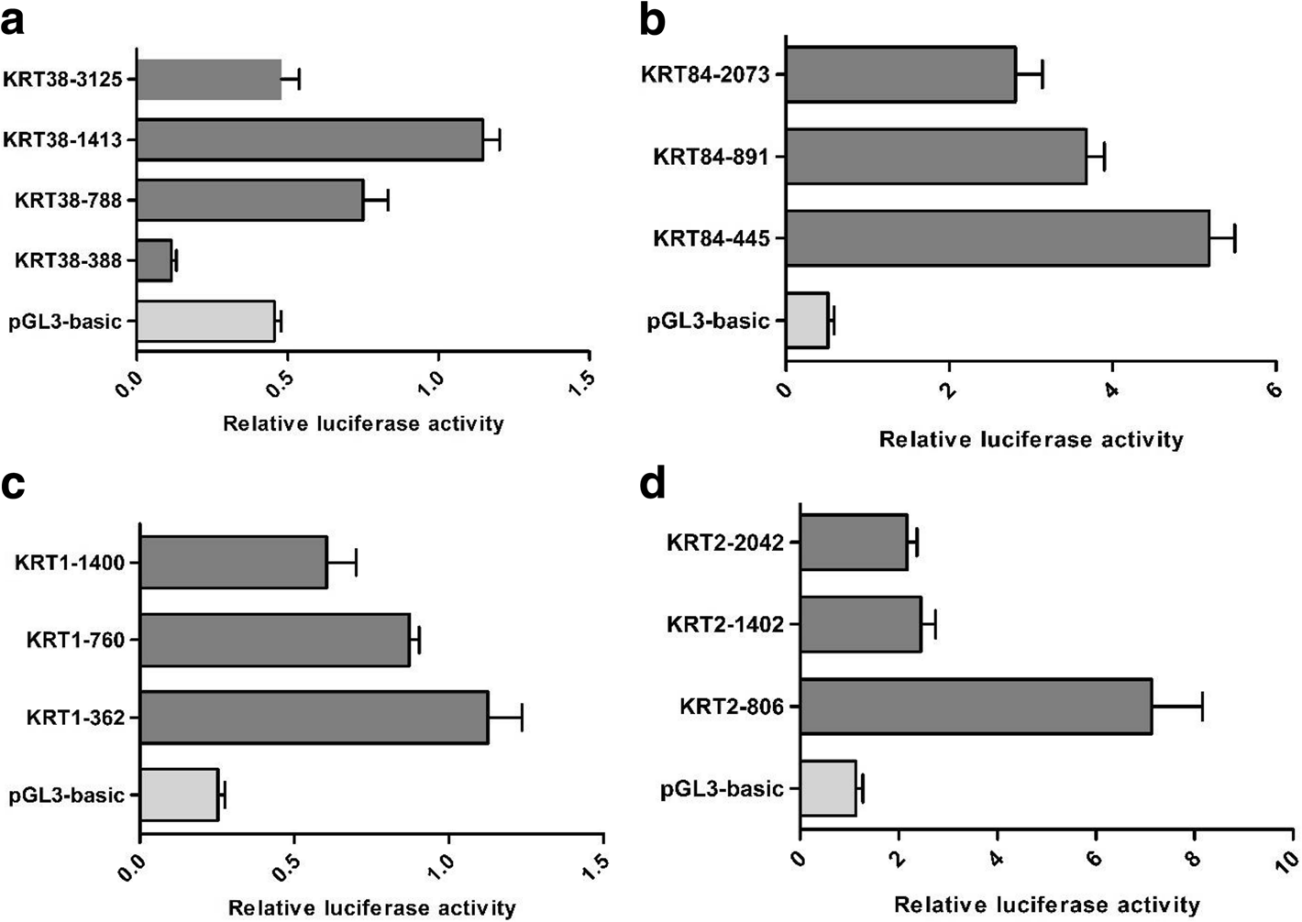
Optimal promoters of *KRT1*, *KRT2*, *KRT38* and *KRT84* were detected using PGL3-basic system. Promoters of *KRT*s with different length were constructed into PGL3-basic system. The length of promoters were shown after hyphen character. The corresponding *KRT* promoter-pGL3 basic vector together with the reference vector pRL-TK were co-transfected into 24-well dermal papilla cells, while the empty vector of PGL3 basic was used as a negative control. All the groups were performed in triplicate

**Fig. 4:**
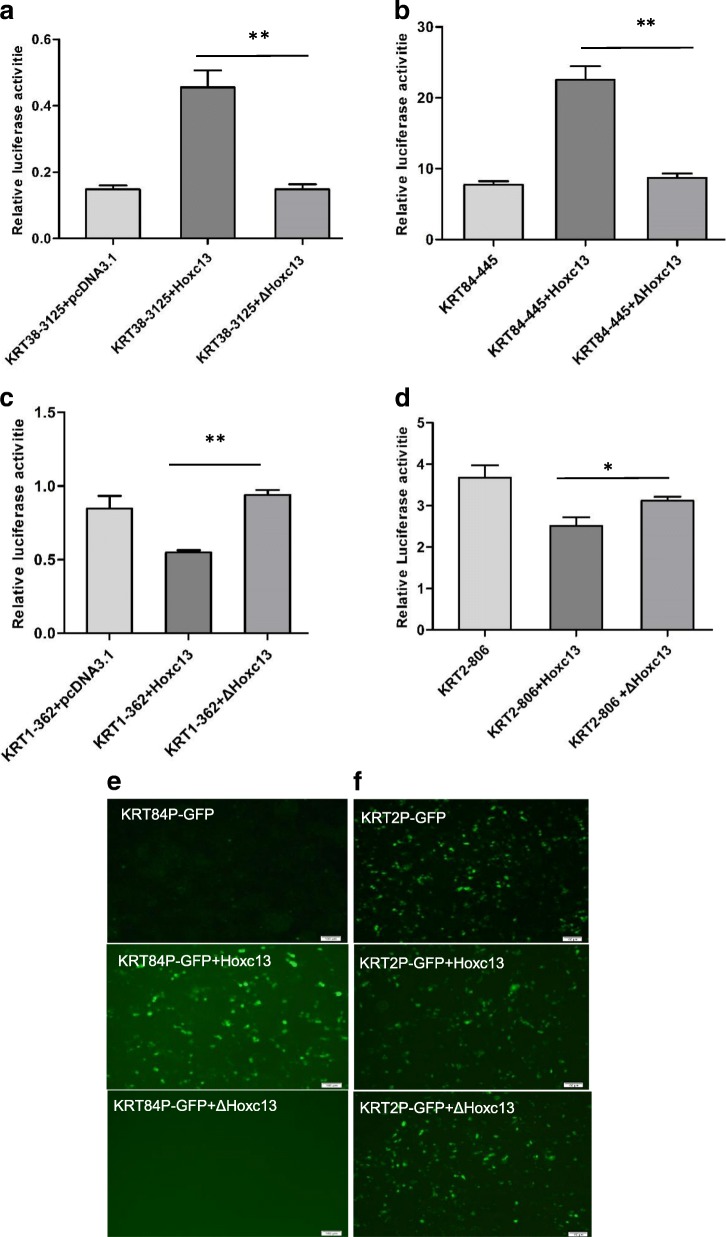
HOXC13 had an inconsistent regulation on the promoters of *KRT1* and *KRT2* compared with *KRT84* and *KRT38*. **a-d** The regulation of HOXC13 on keratin promoters including *KRT1*, *KRT2*, *KRT38* and *KRT84* were detected. The corresponding KRT promoter-pGL3 basic vector, pRL-TK vector together with HOXC13 overexpression vector (wtHOXC13/ΔHOXC13) were co-transfected into 12-well dermal papilla cells, while the vector of ΔHOXC13 was used as a negative control. * P < 0.05, **P < 0.01 (**e-f**) The regulation function of HOXC13 on keratin promoters including *KRT2* and *KRT84* were detected using KRT-promoter-GFP system. In this system, the CMV promoter before GFP was taken place by the promoter of KRT. The corresponding KRT promoter-GFP vector together with HOXC13 overexpression vector (wtHOXC13/ΔHOXC13) were co-transfected into 12-well HEK 293 T cells while the vector of ΔHOXC13 was used as a negative control. All the groups were performed in triplicate. The data was expressed as the mean ± 1 SE (n = 3). * *P* < 0.05, ** *P* < 0.01

For purpose of verifying the regulatory role of HOXC13 on the promoters of keratins, we firstly detected the optimal promoters of selected *KRTs* including *KRT1*, *KRT2*, *KRT38* and *KRT84* using PGL3-basic system. Promoters of *KRTs* with different length were constructed into PGL3-basic system (*KRT* promoter-PGL3 basic). Subsequently, one of these vectors together with the reference vector pRL-TK were transfected into dermal papilla cells. As a result, we found the corresponding optimal promoters for these *KRTs* (Fig. 3). Furtherly, the effect of HOXC13 on keratin promoters was investigated in HEK 293 T cells and dermal papilla cells through co-transfecting HOXC13 overexpression vector, *KRT* promoter-PGL3 basic and pRL-TK vector, while pcDNA 3.1 vector and ΔHOXC13 overexpression vector were used as negative controls. As a result in dermal papilla cells, HOXC13 could up-regulate the promoter activity of *KRT84* and *KRT38* in the group of HOXC13 overexpression compared with the control groups (*P* < 0.05) (Fig. 4a and b), while down-regulated the promoter activity of *KRT1* and *KRT2* (*P* < 0.05) (Fig. 4c and d). The same results were obtained in HEK 293 T cells (Additional file 3: Figure S2). The results indicated HOXC13 had an ambivalent effect on the promoters of different *KRTs*. Interestingly, the promoter activity of *KRT38*-promoter-1413 was not changed when HOXC13 was overexpressed due to lacking the binding site of HOXC13 (Additional file 3: Figure S3). Subsequently, *KRT38*-promoter-3025 with the binding site of HOXC13 was added. Furtherly, *KRT* promoter-GFP vectors were constructed to verify the effect of HOXC13 on the promoters of *KRTs* visually. In this system, the CMV promoter before GFP was taken place by the promoter of KRT. As a result, for the promoter of *KRT84*, the expression of GFP significantly increased in HOXC13 overexpression group than the control groups (*P* < 0.05) (Fig. 3e), while the expression of GFP did not increase for the promoter of *KRT2* (Fig. 3f). The results verified that HOXC13 had an inconsistent regulatory role on the promoters of different *KRTs*.

### The regulation mechanism on HOXC13

**Fig. 5:**
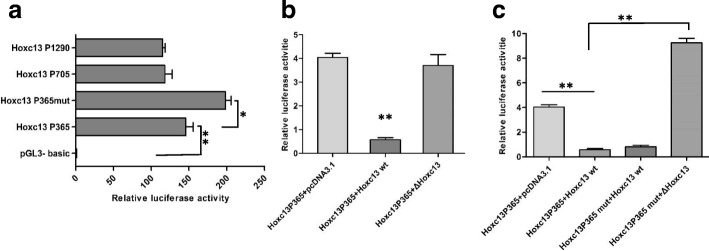
The negative-feedback regulation of *HOXC13* at transcriptional level. **a** Optimal promoter of *HOXC13* was detected using PGL3-basic system in dermal papilla cells. **b–c** The negative-feedback regulation of *HOXC13* at transcriptional level. HOXC13 overexpression vector together with *HOXC13* p365-PGL3 basic vector were transfected into dermal papilla cells, while ΔHOXC13 / HOXC13 mut was used as a negative control. *HOXC13*P365 mut was a negative control to verify the binding site of HOXC13 in the promoter of *HOXC13* itself. The data was expressed as the mean ± 1 SE (*n* = 3). * *P* < 0.05, ** *P* < 0.01

It is essential to understand the regulation mechanism of HOXC13 itself due to its critical function on keratins regulation and hair follicle development. We found a binding site of HOXC13 in its promoter region previously, which indicated the possibility of self-feedback regulation. To verify it, the optimal promoter of *HOXC13* was detected using PGL3 system (Fig. 5a). Subsequently, the corresponding *HOXC13* promoter-PGL 3 basic vector together with HOXC13 overexpression vector were transfected into dermal papilla cells. The result showed that the luciferase activity declined significantly compared with the control groups, which indicated the negative-feedback regulation of *HOXC13* by HOXC13 itself (Fig. 5b). Furtherly, in order to verify the binding site of HOXC13 in the promoter of *HOXC13* itself, the predicted binding site (TT(A/T)ATNPuPu) in the optimal promoter (HOXC13P365) was mutated by introducing a *EcoR* Ι recognition site (HOXC13P365 mut), we found that the luciferase activity was not totally recovered in the group with mutational binding site (HOXC13P365 mut) compared with the wild type binding site (HOXC13P365) (Fig. 5c), which suggested that another binding site of HOXC13 may be existent.

**Fig. 6:**
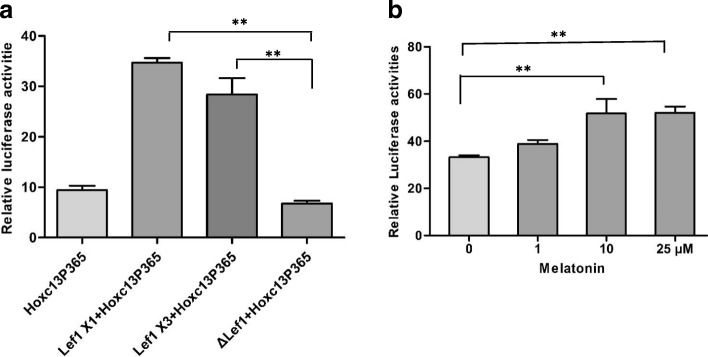
LEF1 and melatonin up-regulated the promoter activity of *HOXC13*. **a** LEF1 promoted the promoter activity of *HOXC13*. LEF1 overexpression vector together with *HOXC13* p365-PGL3 basic vector were transfected into dermal papilla cells. LEF1 X1 and LEF X3 are two spliceosomes of LEF1. Truncated LEF1 (ΔLEF1) was used as a negative control. **b** Melatonin had a positive regulation on the promoter activity of *HOXC13*. Exogenous melatonin with concentration gradient was added into dermal papilla cells together with *HOXC13* promoter-PGL 3 vector. The data was expressed as the mean ± 1 SE (*n* = 3). * *P* < 0.05, ** *P* < 0.01

Meanwhile, we found there were binding sites of LEF1 in the promoter region of *HOXC13*. Using the same method, we detected that the luciferase activity enhanced significantly when LEF1 was overexpressed (LEF1 X1/ LEF1 X3) compared with the control group (Fig. 6a). The resulted revealed that LEF1 could regulate *HOXC13* as a transcription factor by binding the promoter of *HOXC13*.

Melatonin has been reported playing a critical role in cashmere growth and cashmere cycle, while its regulation mechanism was unclear. Exogenous melatonin with concentration gradient (1-25 μM) were added in dermal papilla cells together with *HOXC13* promoter-PGL 3 vector, the result showed that melatonin could significantly increase the luciferase activity under the melatonin concentration of 10 μM and 25 μM, which suggested that melatonin could regulate *HOXC13* through influencing the promoter activity of *HOXC13* (Fig. 6b).

**Fig. 7:**
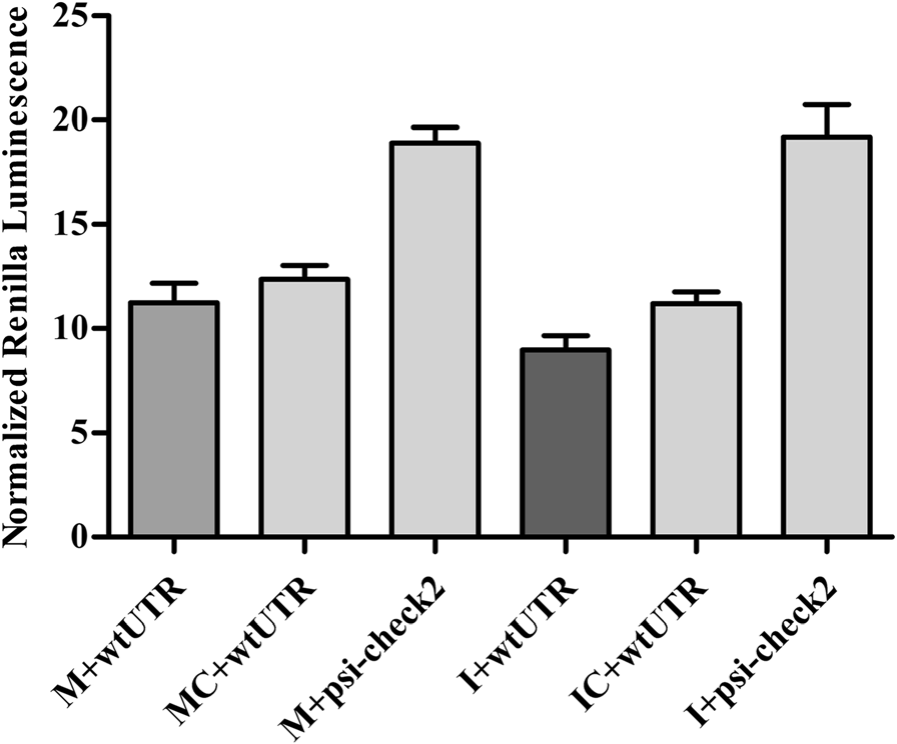
HOXC13 was not the target of chi-miR-200a. Chi-miR-200a mimics/inhibitor/ the corresponding negative control together with HOXC13 3′ UTR vector were co-transfected into HEK 293 T cells, respectively. All the groups were performed in triplicate

At post-transcriptional level, we investigated whether chi-miR-200a could target HOXC13 or not. Mir-200a is a potential conserved miRNA which could target HOXC13 predicted by targetscan. Their interaction was verified in dermal papilla and HEK 293 T cell through dual-luciferase system using miR-200a mimics and inhibitor. As a result, there was no difference of luciferase activity between the groups when miR-200a was overexpressed or inhibited (Additional file 3: Figure S4), which suggested that miR-200a could not regulate HOXC13 (Fig. 7).

**Fig. 8:**
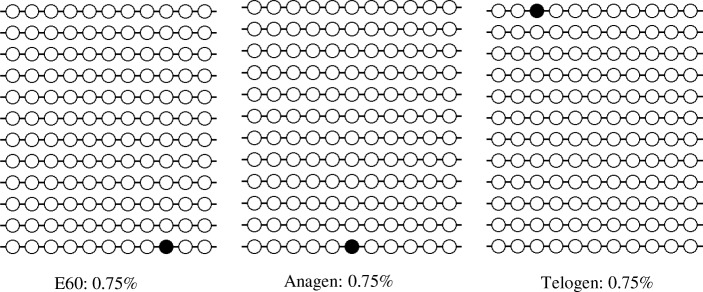
The methylation level of *HOXC13* promoter at different stage including anagen, telogen and E60. The methylation level were detected by BSP and sequencing. Each stage included 3 samples and each sample sequenced 4 clones

At epigenetic level, the methylation level of *HOXC13* promoter at different stage including anagen, telogen and E60 was detected. After applying with bisulfate and sequencing, we found that the methylation level of *HOXC13* promoter had no difference among different stages (Fig. 8).

### Mutations in homeodomain of HOXC13 affect its function

**Fig. 9:**
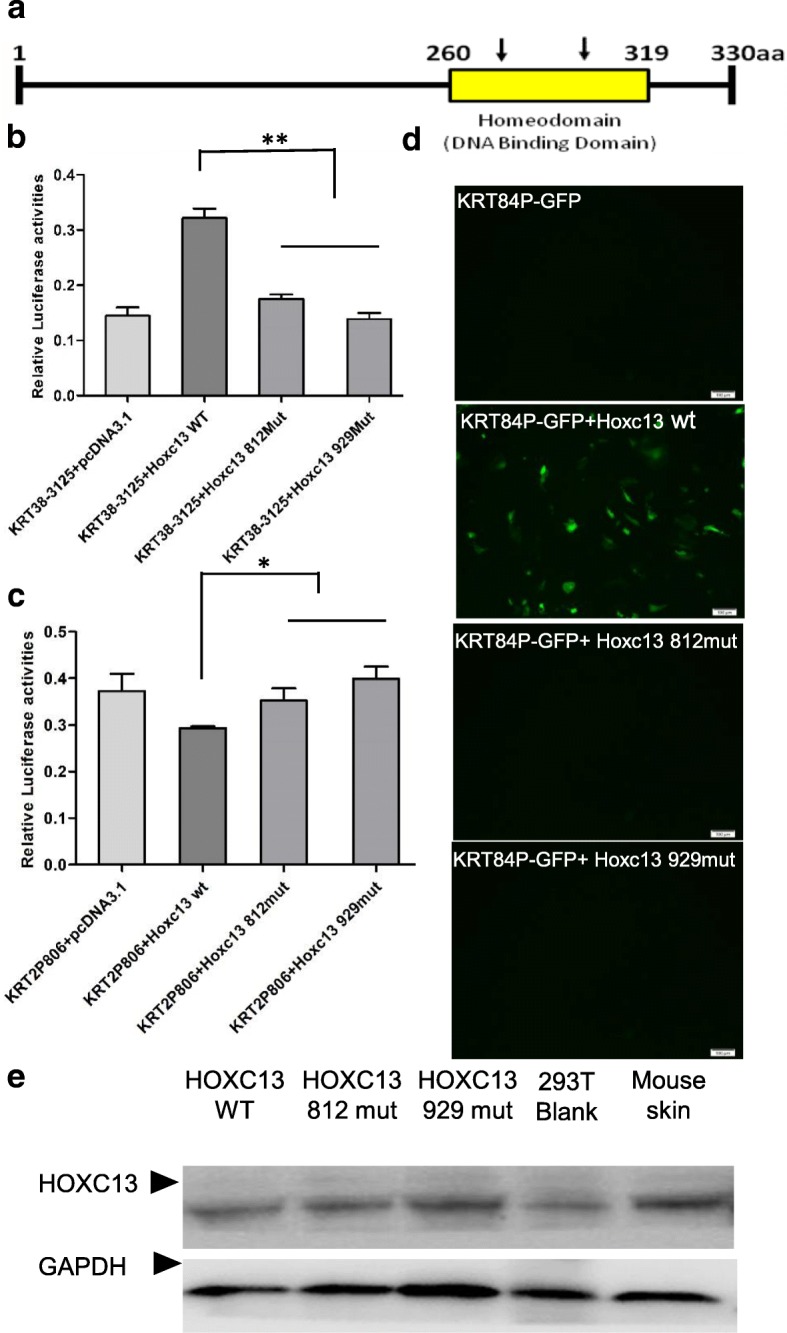
SNPs in the homeodomain of *HOXC13* affect its function on keratins. **a** Representation of *HOXC13* and the position of the mutation (c.812A > G and c.929A > C) within the highly conserved DNA-binding domain. **b-c** SNPs in the homeodomain of *HOXC13* affect its regulation on the promoter of *KRT2* and *KRT38*. The specific *KRT* promoter-PGL3 basic vector together with wild type *HOXC13* overexpression vector or mutational type overexpression vector were transfected into HEK 293 T cells, then the dual luciferase activity were detected. **d** SNPs in the homeodomain of *HOXC13* affect its regulation on the promoter of *KRT84* using KRT-promoter-GFP system in dermal papilla cells. **e** Western blot showed the relative protein level of HOXC13. The total protein was extracted from transfected HEK 293 T cells, blank HEK 293 T cells and skin sample from mouse, while blank cells and the mouse skin sample were used as a positive control

*HOXC13* contains a cluster of 61 amino acids (amino acids 258–318) forming the DNA binding homeodomain, which is conserved across different species (Fig. 9a). In order to investigate the effect of SNPs located in the homeodomain on the function of HOXC13. The mutational HOXC13 overexpression vectors were constructed, which had site specific mutation in the homeodomain. Through co-transfecting HOXC13wt/HOXC13 mut overexpression vector, *KRT38*/*KRT2* promoter-PGL3 basic and pRL-TK vector in HEK 293 T cells, we found that the wild type of HOXC13 could up-regulate the promoter activity of *KRT38* and down-regulated the promoter activity of *KRT2*, while the mutational type of HOXC13 could not (Fig. 9b and c). Through co-transfecting HOXC13wt/HOXC13 mut overexpression vector and *KRT84* promoter-GFP vector in dermal papilla cells, we found that the mutational HOXC13 could not increase the expression of GFP,while wt HOXC13 could significantly increase (Fig. 9d). The results showed that the two SNPs in homeodomain of *HOXC13* affected its function on keratin regulation, thus, caused PHNED. Subsequently, WB was conducted to detect the effect of SNPs on HOXC13 production. The result showed that the mutations had no effect on the protein production (Fig. 9e). Due to the prediction of tertiary structure on HOXC13 (HOXC13wt and HOXC13 mut), we considered that the SNPs may affect the combining capacity between HOXC13 and the keratins (Additional file 3: Figure S5 and S6).

## Discussion

### Spatio-temporal expression and dynamic rearrangement of keratin filament networks

At the onset of anagen, the dermal papilla is close to the hair stem cell located in the follicle bulge. The resting hair bulge stem cells are activated by the signals from dermal papilla. As a result, hair germ envelops the dermal papilla and forms the hair matrix. Transit-amplifying matrix cells proliferate rapidly and promptly undergo a differentiation program to form the inner root sheath and hair shaft [6, 16]. The differentiation products of the temporally regulated processes consist of a large proportion of structural proteins (KRTs and KAPs) within the cells and adhesion proteins between the cells that hold them tightly packed together within the cylindrical-shaped hair [49]. Keratin genes encode the majority of cashmere and hair proteins. The family of keratin genes in human contains 54 functional genes. They have a very distinct expression pattern in different epithelial layers and organs. According the consensus nomenclature for mammalian keratins, the keratin family include three categories: epithelial keratins, hair follicle-specific epithelial keratins, and hair keratins. [18, 50].

Dynamic rearrangement of keratin filament networks is required for epithelial cells to perform cellular processes such as cell migration, differentiation, wound healing and polarization. The mechanisms that regulate keratin network rearrangements and upstream signaling pathways are very diverse and incompletely understood. Several important signaling pathways including Wnt and BMP play an important role in hair differentiation during which keratins are expressed orderly [10, 51]. At post-transcriptional level, keratins are regulated by posttranslational modification (PTMs) located in the head and tail domains as well as in the rod domain, including phosphorylation, O-glycosylation, sumoylation, ubiquitination, acetylation and cysteine oxidation [50]. At transcriptional level, a large number of transcription factors for both positive and negative regulation have been recognized including Gata3, Lef1, Dlx3, Msx2, Foxn1, TCF3 and HOXC13 [24, 52–54], Moreover, neuroendocrine hormones could act as critical transcriptional mediators of hair keratins [55].

### HOXC13 had an inconsistent regulatory role on the promoters of different KRTs

Especially, HOXC13 is essential for hair follicle development and keratins regulation, which had been proved by the hair defects in HOXC13 mutant mice as well as in HOXC13 mutant patients [27, 28, 36]. Subsequently, Tkatchenko revealed that overexpression of HOXC13 in differentiating keratinocytes resulted in downregulation of a novel hair keratin gene cluster [30], while Jave-Suarez showed that HOXC13, but not a homeobox-deleted HOXC13, strongly activated the promoters of the keratin genes [23]. There was no doubt that HOXC13 is involved in keratin regulation. However, the conclusions of the effect of HOXC13 on different keratins were inconsistent. Furthermore, since the previous study focused on specific keratins, whether other keratins regulated by HOXC13 was still unknown. In this study, firstly, we found *HOXC13* had similar expression pattern with the majority of *KRTs* and *KAPs*, and *HOXC13* had higher expression in the period of hair shaft rapidly development, which indicated that HOXC13 had an extensive regulatory role on diverse keratins. Subsequently, we revealed that HOXC13 had an ambivalent effect on the promoters of *KRT38* and *KRT84* compared with *KRT1* and *KRT2* through in vitro experiments. Previous study had revealed an extended 8-bp HOXC13 consensus binding sequence TT(A/T)ATNPuPu. Interestingly, there are multiple binding sites in both *KRT38*, *KRT84* as well as in *KRT1* and *KRT2*, which suggested that there may have other specific binding sequences of HOXC13 for the specific keratin promoter. Otherwise, there may have corepressors and coactivators interacting with HOXC13 to play different role on different keratins, consistent with the model whereby the HOX-PBX complex can act as a repressor or activator of transcription via association with corepressors and coactivators [56, 57]. Besides, we previously found KRT84 was expressed in the trichocyte according the RNA-seq data on secondary follicles of cashmere goat (unpublished data), and KRT38 had been reported having expression in hair medulla of sheep [58], while KRT1 and KRT 2 are epithelial keratins [59], which suggested that HOXC13 may play a different role on keratins with different locations and play a positive role in hair shaft growth.

### The regulation mechanism on HOXC13

More and more functions and mechanisms of HOXC13 had been revealed. However, the regulation mechanism on itself was still unclear. Ansari et al. revealed that mixed lineage leukemia histone methylases played critical roles in estrogen-mediated regulation of HOXC13 [60]. Besides, YAP regulated the expression of HOXC13 in mouse and human oral and skin epithelial tissues [61]. In this study, we investigated its regulation mechanism at transcriptional, post-transcriptional and epigenetic (methylations) levels. As a result, we found chi-miR-200a and methylation had no effect on HOXC13 expression. Interestingly, the negative-feedback regulation of HOXC13 by itself at mRNA level were revealed, which was in correspondence with hair defects in HOXC13 transgenic mice [30]. We also found LEF1 could regulate *HOXC13* by binding the promoter of *HOXC13*. LEF1 is a transcription factor which requires Wnt signaling and stabilized β-catenin to express the hair-specific keratin gene [62]. Our result suggested that there may be interaction and cooperation among Wnt signaling, LEF1 and HOXC13 on keratin regulation. The role of melatonin promoting the Cashmere goat wool and cashmere yield had been demonstrated for decades, while there was still a paucity of knowledge on melatonin mediated hair follicle growth [63]. Our study revealed that melatonin could significantly increase the promoter activity of *HOXC13* under the concentration of 10 μM and 25 μM, which suggested that melatonin could regulate hair follicle development through acting on the promoter of *HOXC13*. However, the exact mechanism need further research.

### Two SNPs in the homeodomain of *HOXC13* affected its function on keratin regulation

In human, *HOXC13* mutations could cause ectodermal dysplasia with complete hair loss and nail dysplasia, which suggested that HOXC13 played a critical role in keratin regulation and hair follicle development again. Most of these mutations (Tyr130*, Leu119Trpfs 20, His68Glnfs*84, Ser135*, 27.6 kb deletion) are nonsense mutations. Interestingly, two missense mutations in the homeodomain of *HOXC13* caused ectodermal dysplasia [36, 37]. However, the mechanism was unclear. Through cloning the mutant gene in vitro, we found that mutational HOXC13 lost its regulation function on keratins while the production of HOXC13 protein was normal. We considered that the SNPs affected the combining capacity between HOXC13 and keratins. In addition to direct target keratins by binding their promoter region, HOXC13 could indirectly regulate keratins by its other target genes. Previous studies had revealed that Foxn1, Foxq1, DSG4, as targets of HOXC13, played a critical role on hair differentiation or keratin regulation [32, 64, 65]. Hence, the SNPs may work through affecting the interactions between HOXC13 and its above mentioned targets.

## Conclusion

In conclusion, HOXC13 had an inconsistent effect on the promoters of different keratins. Two SNPs (c.812A > G and c.929A > C) in the homeodomain of HOXC13 deprived its function on keratin regulation. Besides, the negative-feedback regulation by HOXC13 itself and positive regulation by LEF1 and melatonin on *HOXC13* promoter were revealed. This study will enrich the function of HOXC13 on keratin regulation and contribute to understand the mechanism of hair follicle differentiation.

## Additional files

Additional file 1: The sequences of primers and oligonucleotides. (XLSX 11 kb)

Additional file 2: Gene ontology enrichment result of differentially expressed genes between anagen and telogen. (XLSX 17 kb)

Additional file 3: Supplement figure. (PPT 1024 kb)

## Abbreviations

BSP: bisulphite sequencing polymerase chain reaction
E60: 60^th^d of embryonic period
IHC: immunohistochemistry
KAP: keratin-associated protein
KEGG: Kyoto Encyclopedia of Genes and Genomes
KRT: keratin
PHNED: pure hair and nail ectodermal dysplasia
PTMs: posttranslational modification
qRT-PCR: quantitative real-time PCR
SNP: single nucleotide polymorphism
